# Healthcare scandals and the failings of doctors: Do official inquiries hold the profession to account?

**DOI:** 10.1108/JHOM-04-2018-0126

**Published:** 2019-03-28

**Authors:** Russell Mannion, Huw Davies, Martin Powell, John Blenkinsopp, Ross Millar, Jean McHale, Nick Snowden

**Affiliations:** 1 Health Services Management Centre, University of Birmingham, Birmingham, UK; 2 School of Management, University of St Andrews, St Andrews, UK; 3 Newcastle Business School, Northumbria University, Newcastle upon Tyne, UK; 4 Birmingham Law School, University of Birmingham, Birmingham, UK; 5 Hull Business School, University of Hull, Hull, UK

**Keywords:** Sociology, Doctors, Medical professions, Safety, Quality

## Abstract

**Purpose:**

The purpose of this paper is to explore whether official inquiries are an effective method for holding the medical profession to account for failings in the quality and safety of care.

**Design/methodology/approach:**

Through a review of the theoretical literature on professions and documentary analysis of key public inquiry documents and reports in the UK National Health Service (NHS) the authors examine how the misconduct of doctors can be understood using the metaphor of professional wrongdoing as a product of bad apples, bad barrels or bad cellars.

**Findings:**

The wrongdoing literature tends to present an uncritical assumption of increasing sophistication in analysis, as the focus moves from bad apples (individuals) to bad barrels (organisations) and more latterly to bad cellars (the wider system). This evolution in thinking about wrongdoing is also visible in public inquiries, as analysis and recommendations increasingly tend to emphasise cultural and systematic issues. Yet, while organisational and systemic factors are undoubtedly important, there is a need to keep in sight the role of individuals, for two key reasons. First, there is growing evidence that a small number of doctors may be disproportionately responsible for large numbers of complaints and concerns. Second, there is a risk that the role of individual professionals in drawing attention to wrongdoing is being neglected.

**Originality/value:**

To the best of the authors’ knowledge this is the first theoretical and empirical study specifically exploring the role of NHS inquiries in holding the medical profession to account for failings in professional practice.

## Introduction: professional failures in medicine

Notwithstanding sustained critique (Dingwall and Lewis, 1983; Andrew, 2010) “the professions” remain an enduring and important part of the institutional landscape in modern societies (Noordegraaf, 2012). In most jurisdictions, they are seen as playing an important role in setting standards, rooting out poor practice and disciplining those who transgress against professional (and sometimes legal) norms, and nowhere is this more evident than in medicine (Burau *et al.*, 2009). A defining feature is the claim made for self-regulation. But professions such as medicine sit embedded in wider institutional assemblages, and it is pertinent to ask how are the professions themselves (as institutional arrangements) held to account for this oversight and disciplining role?

Several high-profile scandals involving doctors (and by implication the medical profession) have occurred in the UK over recent decades, and these mirror those found in many other health systems (Lens and Van der Wal, 1997; Braithwaite *et al.*, 2015). The most recent of these (at the time of writing in 2018) is the case of Dr Jane Barton at Gosport War Memorial Hospital, implicated in the premature deaths of over 450 patients through the over-administration of opiate drugs (Gosport Independent Panel, 2018). This followed the previous year’s major story on Ian Paterson (Walshe and Chambers, 2017), a consultant breast surgeon who in 2017 was jailed for 20 years for “wounding with intent” (he invented or exaggerated cancer risks, and over 500 women underwent unnecessary surgery in private and public hospitals with significant iatrogenic harm). Other notable scandals involving doctors in the National Health Service (NHS) include: the case of Harold Shipman, a general practitioner who killed about 250 patients between 1972 and 1998 (Smith, 2004); the clinical incompetence of two paediatric cardiac surgeons resulting in the deaths of up to 35 children at Bristol Royal Infirmary (The Bristol Royal Infirmary Inquiry, 2001); and between 400 and 1,200 patients dying as a result of poor hospital care in Stafford in 2005–2009 (Francis, 2013). Scandals involving doctors (Dixon-Woods *et al.*, 2011), up to and including criminal behaviour (Chamberlain, 2017), are an enduring problem.

In medicine, the day-to-day management of individual miscreants is generally left to their employer or, as a last resort, the professional regulatory and/or disciplinary process. However, wider institutional responses to serious (or widespread) cases of poor care or outright misconduct, especially those that are poorly addressed and/or publicly visible, sometimes include a formal inquiry (Walshe and Higgins, 2002; Walshe, 2003). Indeed, the growing number of such inquiries has been said to suggest dissatisfaction with existing forms of regulation (Goodwin, 2018). A common finding from such public inquiries into medical scandals is that many professionals, often including senior doctors, were aware of the poor care or misconduct, but for one reason or another did not act in effective ways (if, indeed, they acted at all). Moreover, even when staff did raise concerns – informally or formally, perhaps even “blowing the whistle” to outside authorities – they were often either ignored or responded to inappropriately by their host organisation and/or other agencies in the system (Vandekerckhove and Tsahuridu, 2010).

In this paper, we develop a multi-level analysis of poor practice and misconduct involving doctors in the UK. Drawing loosely on the analytical framework developed by Kish-Gephart *et al.* (2010) and extended by Muzio *et al.* (2016), we explore how problems (and solutions) in managing professional failings in healthcare can arise at three levels. First, problems can arise at the level of the individual doctor, where personal shortcomings – from incompetence to criminality – are seen as examples of “bad apple” professionals. Second, the organisational setting can be diagnosed as inimical to good practice, with the usual invocation of “culture” as the culprit (“bad barrels”, to continue the metaphor). Third, the overall health system can be implicated, identifying failings in various constituent parts, and the relationships and coordination between them, as well as in the overarching policy framings and directives (a case of “bad cellars”). In addition, we contend (see later) that the overall nurturing of professional attitudes and practices through initial professional education and training, as well as through subsequent post-qualification specialisation and extra-organisational enculturation, extend the metaphor: these professional processes can be seen as creating an “orchard” where new professionals are “grown”, perhaps even in ways that make professional failings (and institutional failure to tackle these failings) more likely.

These levels are, of course, connected: the overall system-level arrangements condition and inform the organisational and professional context, and these in turn shape and constrain individual behaviours. At the same time, tacit knowledge and patterns of practice in clinical communities accrete and solidify to inform higher-level assessments about acceptable norms. That is, new institutional logics (Thornton *et al.*, 2012) can form and solidify at multiple levels, being given additional weight when adopted by parts of the formal professional architecture.

In seminal work from nearly thirty years ago, Donald Light articulated the concept of “countervailing power”, the dynamics that ensure when “one party has accumulated such power that it prompts other parties to muster their forces and attempt to control the first” (Light, 1991). While such deliberative attempts to reassert control are important, we can also see that power, its consolidation and its expression, may be impacted less directly and less deliberately – as by-products or unintended consequences of other changes, for example. Thus, it is important to see that these wider dynamics have the potential to exacerbate existing risks and to create new vulnerabilities, such that the protective arrangements against wrongdoing require constant attention (e.g. Muzio *et al.*, 2016). Public inquiries into professional failings can thus be seen as having an important role (both instrumental and symbolic) in promoting or restraining the formation and/or embedding of new institutional logics, and supporting (or impeding) the reform of regulatory processes, with consequences for the restraint, surfacing and addressing of future professional failings.

Our focus then, in this analysis, is on exploring whether and how the medical profession itself is being held to account and encouraged towards appropriate reform as a consequence of public inquiries into individual professional failings or wrongdoing. Such dynamics are complex and problematic because the self-regulatory nature of the profession contains within it the logic that the professional should not only hold individual doctors to account, but should also hold itself to account for its regulatory processes (so-called “new professionalism” Evetts, 2009; Noordegraaf, 2012). And yet the profession is not immune from broader influence and even statutory control, so it is reasonable to explore how such influence is expressed and asserted.

We begin by exploring the (publicly financed and often publicly owned) health system within which the overwhelming majority of UK doctors work, the NHS, and outline the complex institutional arrangements for ensuring safe, high-quality care. From this we can identify the crucial role played by “stories from the front-line” – from staff, patients and relatives – in surfacing concerns, failures and misconduct. However, we also note the systemic features that attenuate or block such accounts, including professional solidarity and anxieties over repercussions. This leads us to explore the various ways that the professions – especially the medical profession – have evolved historically, and how they have been construed sociologically, as a means of beginning to examine the challenges of gaining clear sight on the reform needs of professional oversight. Finally, we explore – from an empirical documentary review – the relative failure of formal inquiries to address the wider needs of system change if parts of the “cellar” (and, indeed, the “orchard”) are to see the reform that they need. Specifically, we highlight the need for the medical profession itself to become better equipped at dealing with the individual wrongdoing for which new dynamics have emerged.

## The NHS health system (a hallowed cellar?)

Established in 1948, the NHS is one of the largest and most complex organisations in the world, employing over 1.4 million full-time equivalent staff, including more than 150,000 hospital doctors and 40,000 general practitioners. In England, the NHS is formally accountable to parliament through the Secretary of State for Health (other jurisdictions in the UK have their own arrangements under devolved settlements). In terms of structure, the NHS is made up of a complex “hierarchy and network” of sub-organisations, each legally a separate employer, and each with different responsibilities and varied inter-organisational contractual and reporting arrangements. In addition, a plethora of regulatory and oversight agencies exist to assess and ensure high-quality care, with many reforms and structural reorganisations of these agencies over recent decades. Alongside these statutory and delivery organisations sit the professional bodies, with diverse arrangements for doctors, nurses, midwives and allied health professionals. For doctors specifically, organisation, oversight and promotion of their professional roles is arranged through three main strands: there are various “Royal Colleges” (RCs); (arranged by specialty and sub-specialty, focussing on post-qualification education and accreditation); there is the General Medical Council (GMC); (setting standards, especially around education and initial qualification; arranging registration and licensing; and dealing with concerns about malpractice); and there is the British Medical Association (BMA); (an advocacy organisation for the profession, essentially “the doctors” Trade Union’).

Since its inception, the NHS has been the subject of successive waves of structural reorganisation and reform. Traditionally dominated by medical professionalism (Dixon-Woods *et al.*, 2011), the NHS has, from the 1980s onwards, been subject to more extensive managerial control and marketised restructuring as part of wider New Public Management trends (Exworthy *et al.*, 2016). These include such changes as the reform of professional regulation and oversight, including more extensive methods of measuring and assessing organisational and individual performance, and the use of financial incentives to drive performance improvements. This has led to a range of market, corporate and professional logics being accommodated, blended and contested at the local organisational level (Currie and Spyridonidis, 2016; McDonald *et al.*, 2013,) and claims that the medical profession is in flux, being systematically de-professionalised, proletarianised and restratified (Ritzer and Walcak, 1988; McKinlay, 1982; Waring, 2014 and see expanded discussion below).

But the bare bones of this account do not convey the almost hallowed role that the NHS plays in the UK national psyche (e.g. the prominence of the NHS in the opening ceremony of the 2012 London Olympics showcasing the UK to the world). This hallowed role has enormous contextual significance when seeking to understand the dynamics of professional failings and the hesitant steps towards professional reform. Notwithstanding periodic and very public crises around failings, the NHS remains the most trusted of British institutions (British Social Attitudes Survey, 2016), and doctors remain the most trusted of professions. Indeed, the NHS retains huge public support and politicians of all ideologies struggle to manage its reform, despite its evident social and economic importance and despite much evidence of the need for that reform (Exworthy *et al.*, 2016).

Curiously, while doctors remain most trusted, and most influential in directing or resisting reforms, they have always remained a little outside of (or semi-detached from) the organisations that make up the NHS. From its inception in 1948, doctors had to be coerced/bribed into the new structure (Bevan’s infamous quote that he “stuffed their mouths with gold” Klein, 2013), and even now most family doctors (GPs) remain as independent contractors, not salaried employees of the NHS. More recent work on the relations between doctors and managers in hospitals (Powell and Davies, 2016) suggests that there is an increasing gulf between doctors and the managerial structures that oversee them, and that even where good relations exist there are fragilities that can prompt disengagement.

## Surfacing professional errors and wrongdoing (finding bad apples)

Professional work often requires judgement based on hard-to-codify knowledge, alongside skills and expertise that may be imperfectly expressed, in situations of risk and uncertainty. Moreover, the professions themselves may resist codification as a bulwark against de-professionalisation (Timmermans and Berg, 1997). It is no surprise then that identifying professional errors and wrongdoing is difficult and fraught with ambiguity. One influential categorisation of transgressions identifies “slips”, “lapses”, “mistakes” and “violations”, each of which involves differing degrees of culpability (Reason, 2000). The situation is further complicated as rules-based policies, procedures and standards have become commonplace: “workarounds” may be required to deal with anomalies or contradictions between policies, or even just to ensure smooth workflow (Debono *et al.*, 2012). This introduces the challenge of judging appropriate and judicious violations from unnecessary transgressions, calling into question not just actions and competencies, but also motives. Many opportunities to raise issues about quality or safety occur quite routinely and are deeply embedded in clinical work; and diverse informal strategies of correction are often effective (Tarrant *et al.*, 2017). Yet “calling out” errors and wrongdoing remains a risky business, especially in professionalised and hierarchical organisations, where power is never far from the surface (Currie *et al.*, 2012; Waring, 2014). Indeed, the questioning of a fellow doctor’s skill or judgement has, until recently, been explicitly proscribed in professional codes of conduct as itself an example of misconduct.

Unsurprisingly then, national staff surveys suggest widespread disquiet about raising concerns about poor or unsafe patient care in the NHS (NHS National Staff Survey, 2015/2016). There is a perception among health professionals that they may be ostracised, bullied, or put their careers at risk if they were to raise concerns about colleagues (MPS, 2012). The reality of “working for the NHS” means that raising concerns or “blowing the whistle” on professional misconduct in any one NHS organisation risks not only professional ostracism and local organisational retaliation, but also an effective “blacklisting” from employment elsewhere in the service. Despite some expansion in private healthcare, the NHS remains the major employer for most healthcare professionals, so being excluded from the NHS almost amounts to being excluded from employment, at least within the UK. A related point is that in an NHS densely linked by formal and informal networks, staff may have concerns about their ability to find adequate confidential support and guidance, with some former whistleblowers recalling being shocked at how little support they received from professional bodies, regulators or even trades unions (Francis, 2013).

Taken together then, there are powerful reasons for professionals not seeing, believing or acting upon evidence of wrongdoing amongst their peers; and there are significant impediments to others doing so. Managers and nurses, for example, may lack the knowledge and status to query doctors’ behaviour, and may themselves suffer from similar risks to their organisational and professional life as doctors; patients, relatives and carers may be impeded through lack of specialist knowledge, confidence or power.

Whatever these challenges, finding – and dealing effectively with – “bad apples” is especially important as evidence grows that small numbers of professionals may be disproportionately responsible for large numbers of complaints and concerns (Bismark *et al.*, 2013). As a result, the internal workings of the profession – and in particular, its capacity to support and protect those at the frontline who do observe and report potential problems – becomes paramount, as does an examination of the mechanisms – such as inquiries – that can be used to shape these arrangements.

## The medical profession (both barrel and orchard?)

The structures underpinning the profession of medicine in the UK date back to the nineteenth century. The Provincial Medical and Surgical Society was formed in 1832, becoming the BMA in 1855, which continues to this day. However, political pressure for reform eventually culminated in the 1858 Medical Act, which marks the modern period of medical professionalism in the UK. The Act created what eventually became the GMC and entered medicine into a regulatory agreement with the state.

Doctors receive much of their socialisation and collegial etiquette through their professional training and engagements rather than as a consequence of their organisational setting (Becker *et al.*, 1961; McDonald, 2014). Moreover, in the UK certainly, doctors – even more so than other professional elites such as law and journalism – are drawn disproportionately from the more advantaged strata of society: just 4 per cent of UK doctors come from working class backgrounds, and 80 per cent of UK medical school applicants come from around only 20 per cent of all schools, predominantly independent (i.e. fee-paying) or grammar schools (Social Mobility Commission, 2016). It hardly needs saying of course that despite any NHS and/or organisational ambivalence noted earlier, the medical profession has succeeded in extracting consistently high societal rents (Klein, 2013).

The various branches of the medical profession noted above (the RCs, the GMC and the BMA) provide collective control over education, standards and licensure, and important venues for occupational socialisation and the encouragement of professional collegiality (Aveling *et al.*, 2016). Through registration with the GMC, the profession has (since the mid-nineteenth century) possessed an occupational monopoly (and hence powerful incentives for individual doctors to be appropriately socialised), with “registration” required with the GMC for any doctor to practice in the UK. The self-regulatory “collegial model” also creates a fiduciary responsibility to patients and the general public with the profession as a whole acting as guarantor for the performance and conduct of each of its members.

From its beginning, the Council had the power to remove individuals from the register as a penalty for “serious professional misconduct”, though such cases were usually sexual in nature and until the 1980s the Council took a very narrow view of its role, being generally reluctant to pursue allegations of professional incompetence. As a result, serious and culpable behaviour, including clinical incompetence, sexual wrongdoing and even deliberate patient harm were known to have occurred, often unchecked. Even after significant reforms in the 1990s, referring to “a new professionalism […] fundamentally different from the past” (Irvine, 2001), a recent review of GMC activity (2005–2015) showed that “no doctor was barred from practising medicine for serious violent and sex offences, including rape […] manslaughter and domestic violence” (Chamberlain, 2017). As Dixon-Woods *et al.* (2011) argue, “unravelling the puzzle of why the profession did not act to deal with individuals who posed such a serious threat to its own legitimacy requires recognising that the system imperative to engage in monitoring and correction of deviant behaviour was in conflict with the social imperatives for collegial cooperation”. If, as the old adage has it, “the apple rarely falls far from the tree”, then the custodians of the orchard may have been persuaded that socialisation processes alone would be sufficient to minimise misconduct.

## Theorising the profession

Since the late 1950s, several conceptual approaches to the professions can be discerned, with the medical profession often being the example of choice for sociological theorising. The first systematic study viewed the professions as central to the smooth functioning and stability of modern societies (Parsons, 1951). From this perspective, the professions are assumed to exercise an important benign social role by virtue of their esoteric knowledge and expertise that are used for the benefit of society. Nevertheless, this “functionalist” approach acknowledges that the medical profession’s tacit knowledge and technical expertise opens up the potential for abuse. To prevent this, and in return for being granted an elevated social position, autonomy, high financial rewards and legal protection, the profession is expected to submerge its self-interest and enter into an implicit social contract which ensures that their privileged position is used for the benefit of society.

From the late 1960s, growing dissatisfaction with functionalist explanations gave way to more critical perspectives. The emerging “power perspective” emphasised the role of professional self-interest rather than selfless altruism, and sought to explicate the political and social processes by which the professions attain and maintain their privileged position in society (Larson, 1977). Here the professions are seen to rely upon their asserted ethical standards in order to justify self-regulation and to defend their territory. Yet, as Freidson (1970) notes, the medical profession may deceive others (and themselves) into believing that they are acting in the public and client interest, while abusing their authority for collective and individual gain.

Notions of misconduct and wrongdoing are central to both the functionalist and the power perspectives on the professions. In the functionalist view, professional codes simply preclude misconduct, with such behaviours being both deviant and aberrant. When misconduct does arise, it is blamed on the behaviour of “bad apples” or rogue individuals who violate the norms and moral code of their profession (Muzio *et al.*, 2016). In the power perspective, the professions are not assumed to have any special moral commitment to the public good and are viewed more as self-organising cabals that use their institutionalised protection from competitive forces to engage in self-serving behaviour. Medical misconduct in this sense is consistent with a “bad barrel” hypothesis (Kish-Gephart *et al.*, 2010; Muzio *et al.*, 2016) as it arises from the way in which the medical profession is organised; but these arrangements are embedded in a wider socio-political context and so there are also elements of the “bad cellar” hypothesis. Moreover, dynamic shifts in these arrangements may expose new vulnerabilities or exacerbate existing ones (Muzio *et al.*, 2016). For example, new financial or reputational incentives may change clinical behaviour in unexpected and unwarranted ways: the extension of clinical performance measurement systems has undoubtedly incentivised a range of dysfunctional behaviours, for example (Mannion and Braithwaite, 2012). Indeed, any shift in the organisational and regulatory environment may give rise to a wide array of unintended consequences, and so provide scope for new forms of failure and wrongdoing.

While the power perspective continues to be influential, its core argument – that the medical profession is a self-interested cabal – has attracted considerable critique. One line of argument suggests that the powers of the medical profession are constantly being eroded, a process known as proletarianisation. This thesis highlights the potential for expert work, including medical work, to be subject to the codification and standardisation that allows more bureaucratic control in the name of controlling costs and minimising risks (Timmermans and Berg, 1997). A second line of argument, linked to some of these trends (known as the deprofessionalisation thesis), focusses on the fact that attitudes to traditional forms of authority are changing. In particular, it notes a decline in the public trust of public institutions (Noordegraaf, 2012), with the public increasingly expecting their public services – including healthcare – to operate in transparent and accountable ways. These arguments combined raise serious questions about the validity of viewing the medical professions as simply an effective exercise in economic self-interest – other forces are at work that constrain and direct professional work to broader ends.

A third challenge is that – rather than undergoing a period of decline as supposed by the proletarianisation and deprofessionalisation theses – medicine is instead experiencing a process of “restratification” that is sustaining medical privilege and power, albeit in smaller and tighter groupings. This argument asserts that the medical profession is becoming (re-) stratified into distinct “elite” and “rank and file” roles, with differential power, rewards, prospects and status (Noordegraaf, 2012). In this way, medical elites increasingly subject “ordinary” practitioners to greater peer surveillance and control as they seek to maintain collective regulatory privileges, albeit in a new and more transparent form.

In this complex and changing context, it has been argued that a more dynamic approach to professional misconduct is needed: an ecological perspective (Abbott 1998, 2005), consistent with a “bad cellars” metaphor, in which misconduct is understood in the fast-changing political and economic contexts that are morphing the traditional institutional arrangements of the medical professions and that are impacting on their powers to promote and regulate appropriate professional behaviour (Muzio *et al.*, 2016). Here the medical profession is conceived as part of a broader ecology with adjacent institutions – reflecting that the medical profession is constrained, supported and generally affected by the moves of social actors adjacent to them and with whom they regularly interact (what Abbott, 2005 refers to as “linked ecologies”). In this context, professional misconduct (and also, potentially, its surfacing and its remedy) is seen to arise from the re-drawing of boundaries and relationships within and between professional ecologies (e.g. between doctors and nurses, and between doctors and managers). A particular concern is that boundary changes may undermine existing oversight regimes, fuel conflicts of interest, create regulatory blind-spots and so generate opportunities for malpractice and other types of wrongdoing (Muzio *et al.*, 2016).

Moreover, the ecological perspective outlined above cannot confine itself to competing professional arrangements but must be cast wide enough to encompass government pronouncements, legislative actions and regulatory shifts. Interactions between these may produce unexpected tensions and contradictions. For example, the recent case of a Dr Bawa-Garba who was investigated after the death of a child under her care highlights some of these. This case was first taken through the courts (where Dr Bawa-Garba was convicted of manslaughter through gross negligence), but when taken before the professional regulation mechanisms of the GMC the doctor was merely suspended for 12 months, with the GMC arguing that “erasure [from The Medical Register] would be disproportionate”. This decision was subsequently appealed to the high court. The repercussions of this case demonstrate some of the tensions between legal and regulatory remedies as well as with professional codes of practice and statutory duties such as the newly introduced “duty of candour”. An ecological perspective must give wide enough purview to capture such interactions.

But how can deleterious aspects of the “orchard”, “cellar” and “barrel(s)” be identified, analysed and remedied? And how successful in doing so are the current arrangements for looking at “the wider picture” surrounding high-profile cases of wrongdoing in healthcare? In particular, have formal public inquiries into professional wrongdoing in the NHS served us well to-date in providing clear sight of these ecological failings and their possible remedy?

## NHS Inquiries (rooting around in the cellar)

In the UK, formal public inquiries (either statutory or non-statutory) are a long-established part of the institutional arrangements for relieving political pressure, restoring public confidence and encouraging system reform. Inquiries typically involve “a retrospective examination of events or circumstances […] to find out what happened, understand why, and learn from the experiences of all those involved” (Walshe and Higgins, 2002, p. 851). NHS-specific inquiries are usually initiated by the Secretary of State for Health, and even then, often only after long and loud public concern evidenced through media pressure. Such inquiries may have legal power to call witnesses and take expert testimony. The primary output generated by an inquiry is its official report, and the most influential component of that report is the set of recommendations detailing key learning points and desired future actions (Williams and Kevern, 2016). Relevant agencies, including government, statutory bodies and both professional and lay associations, may then themselves make formal representations in response.

In an analysis of NHS inquiries since 1967, Lehane (2015) identified three main reasons for their enactment: in response to major failure or scandal; as proactive scrutiny of nascent areas of concern; or as a means to assuage loss of public confidence (of course, two or more of these may pertain). Similarly, Walshe (2003) identifies six purposes for formal inquiries: to establish “the facts” around service failures and encourage learning from events; but also: to promote cathartic change; provide reassurance; hold those responsible to account; and to address the political aspects of the circumstances of the inquiry. The diversity of purposes identified by these authors point to important tensions: the broader symbolic and ritualistic tasks that inquiries perform may impede the more instrumental task of implementing effective change in institutional arrangements, and this may be one reason why formal NHS inquiries usually bring very little identifiable change (Timmins, 2013).

## A textual analysis of recent NHS inquiries

While recognising the multiple, complex and often ambiguous nature of inquiry purposes, we can nonetheless examine them for their degree of engagement with the surfacing and addressing of professional wrongdoing. In particular, it may be helpful to examine the implicit assumptions made about professionals, professional wrongdoing and professional oversight, that are buried in the inquiries terms of reference, modes of practice, articulation of events and recommendations. Thus, here we present the findings of an analysis of all the major, formal NHS Inquiries into poor doctoring since 2000, dating back to the Bristol Royal Infirmary (Kennedy, 2001) through Ayling (2004), Neale (2004), Kerr/Haslam (2005), Shipman (2005), Francis (2010), Francis (2013) to the Francis (2015) “Freedom to Speak Up Review”. The inquiry reports (and formal Government and other institutional responses) were searched (electronically) through keywords such as “Whistle*” (raising) “Concern”, “Speak*” ‘Bully*, “Victim*”, “Intimidat*”, “Reprisal”, “Silen*” and “Fear” (the asterisk (*) represents “wildcard” letters, so that, for example, “Whistle*” would capture whistle-blower and whistleblowing). The sections revealed by these searches (and surrounding paragraphs, together with any signposted links) were then explored in more detail, building up a detailed picture of how wrongdoing was identified and dealt with, alongside recommendations for better handling of this in future.

A number of common issues arise from this textual analysis of the inquiries and responses, and it is to these that we now turn. Table I highlights and summarises key observations from each of the inquiries covered. Separate columns in this table identify the nature of the individual wrongdoing (bad apple behaviour), any critique of the organisational setting (bad barrel features) and wider criticism of the health system (bad cellar). What follows below is a distillation of the key points arising from these inquiries in relation to individual wrongdoing and the calling out of that.

## System failures, rather than individual shortcomings

It was notable that most inquiry accounts focussed on the local organisational arrangements and/or the broader system-level arrangements, rather than on the people located at the sharp end of failure; that is, barrels and cellars generally received more attention than bad apples, even when the inquiry was specifically focussed on the wrongdoing of one or more individuals. In doing so, the inquiry reports routinely emphasise structural and collective, rather than agency or individual, explanations (see Table I). One exception to this was the Kerr/Haslam Report (2005), which highlighted whistleblowing in the case of Kerr. As the counsel for the patients put it to the inquiry: “for the main part, we do not say these are system failures, they are personality failures” (Vol 2, pp. 801-802). However, such pinning of responsibility for failure on individual actions or inactions was very unusual in the inquiries reviewed, which very much offered up explanations based on systemic failings, typically at an organisational level.

### Cultural change over legal safeguards

Likely because of the emphasis on “system failings”, the most consistent remedy drawn out from inquiry recommendations has been cultural reform and renewal rather than legal safeguards or deeper rethinking about the safeguarding role of the professions. In focussing on problematic cultures in this way, inquiries can be seen to “distribute a collective responsibility for healthcare failures” (Goodwin, 2018).

Inquiries since Kennedy (2001) have consistently argued in favour of “culture change” (without specifying in any detail how this was to be achieved, Davies and Mannion, 2013). For example, Francis (2010) pointed to the problem of organisational culture, and considered that “the most important factor in changing this will not be a new system or policy of protection for whistle-blowers, but the fostering of a culture of openness, self-criticism and teamwork”. Without a positive culture (it was claimed), it would never be easy to raise concerns: Francis considered that whistleblowing was only necessary because of the absence of systems and a culture accepted by all staff that is receptive to internal reporting of concerns. “Therefore, the solution lies in creating the right culture, not in focussing on improvements to whistleblowing legislation, important though such protection is” (p. 242). Similar points are made by the government in response: while progress on Francis’ 290 recommendations was said to be necessary, “perhaps the most important point is […] the ongoing need to change the culture in the NHS to one of patient-centred, continual improvement in care and safety”. That cultural change is as unproblematic as such pronouncements seem to suppose has, however, received some considerable challenge (Davies and Mannion, 2013).

## Raising concerns, blowing the whistle

The inquiries reviewed did not generally concern themselves with successful day-to-day error spotting and correction within organisations; they were more concerned to identify “failures to act” that led to on-going failings and wrongdoing, and with the wider system failures that saw warnings, including whistleblowing, go unheeded. However, few inquiries tried to define the concept; and indeed, the Shipman Inquiry tried to avoid using the expression “whistleblowing” at all (2005, p. 319). Francis (2015) considered replacing the term, before presenting a broad definition: as “a person who raises concerns in the public interest”. Yet many persons who raise concerns do not necessarily, at the time of raising the concerns, see themselves as whistleblowers. They are likely to come to regard themselves as whistleblowers only if they suffer detriment as a result of raising the concerns or if no action is taken on their concerns (p. 2). The Shipman Inquiry considered that none of those persons who raised concerns were “whistleblowers” *per se*, as they did not work within the same organisation as Shipman, (p. 318). Similarly, The Bristol Inquiry did not appear to recognise the anaesthetist who raised concerns as a whistleblower, stating that had the Public Interest Disclosure Act been in force it would not have applied (2001, p. 160).

That there should be so much uncertainty and disquiet about speaking out should not come as a surprise. Local discursive practices (e.g. on the nature of success, failure, risk and performance) and local operational contingencies (such as resource constraints, service rivalries, and stakeholder pressure) will have a powerful influence on the willingness of employees to raise concerns and the ability and willingness of employers to respond appropriately. However, the chair of the Shipman Inquiry, Janet Smith, stated that “I believe that the willingness of one healthcare professional to take responsibility for raising concerns about the conduct, performance or health of another could make a greater potential contribution to patient safety than any other single factor” (2005, p. 23) (subsequently cited in Hooper, 2015, p. 2 and Francis, 2015). Similarly, as Niall Dickson, CEO of the GMC put it in 2012: “The eyes and ears of health professionals are often the most valuable means of protecting patients and ensuring high quality care” (in Hooper, 2015, p. 20). Yet of some 820 recommendations across all of the inquiries reviewed we found only eight that were directly concerned with whistleblowing; and even in the Shipman Inquiry, which stressed the role of health professionals in raising concerns, we found only three of its 190 recommendations focussed on whistleblowing.

### Misplaced optimism

Evident from these inquiry reports is a high degree of re-inventing the wheel (Powell and Mannion, 2016). The clearest example of this is the repeated identification of culture as both culprit and solution to periodic failings in the quality of care in the NHS (discussed above). A further example is the term “duty of candour” (i.e. a requirement, either in terms of professional codes or even in statute, for professionals to speak out when they notice failure or wrongdoing), which appeared in Kennedy (2001) through Kerr/Haslam (2005) to Francis (2013), Dalton and Williams (2014) and DH. Similarly, there has been a repeated stress on re-issuing policies and guidelines, despite scant evidence that these have much effect (Shipman, 2005; Kerr/Haslam, 2005). Indeed, Francis (2013, p. 280) noted that the Mid Staffordshire Trust had actually had a policy on whistleblowing since 2001, but events exposed the “hollowness” of that policy.

Overall, many of the reports appear to be somewhat optimistic that “things are getting better”: that institutions, policies and procedures are in place that will not allow earlier problems to reoccur (e.g. Ayling inquiry; Shipman inquiry). Governments tend to argue that “much has changed” since the incidents took place, and that remedial policies have been put into place. Not all commentators would agree, however, and the treatment of whistle-blowers in particular remains highly controversial, even from within Parliament (O’Dowd, 2015). Thus, while there have been some positive changes, there must be a concern that the changes seen are not sufficiently comprehensive enough, or dynamic enough, to repair the weaknesses of professional regulatory control. Indeed, the impression gained across these inquiries is one of missed opportunities and failed learning: the word “hindsight” was used 456 times in the testimony to the Francis public inquiry. In terms of whistleblowing, speaking up, and addressing professional wrongdoing, the NHS appears to have much to learn before becoming a learning organisation (Davies and Nutley, 2000; Harvey *et al.*, 2015). Moreover, the regulatory control of the medical profession cannot yet be said to be fully functional, with reform through inquiries having been scant.

## Concluding remarks

Our theoretical and empirical analysis has several implications for understanding and addressing professional failures as well as for dealing with weaknesses in professional regulatory systems. First, seeking out “bad apples” is core work for the professions if their claims to self-regulation are to be taken seriously – so surfacing and dealing with “bad apple” doctors is paramount. When the discourse promulgated through inquiries backgrounds “individual blame” just as it foregrounds “systems failure”, something has been lost. The move to emphasise “just cultures” (where the need for learning from an open assessment of errors is balanced with the need to hold individuals to account for those errors) is a reflection of a need for rebalancing being taken up by other parts of the ecology. Thus, while there is an important role in preventing, and addressing culpable failures at the local level, there is also a need to develop accountability systems that reflect the dynamic and recursive nature of the duality of structure and agency in shaping and driving professional behaviour in healthcare settings (Aveling *et al.*, 2016).

Second, we can also consider (from the power perspective on professions) that the intrinsic self-serving nature of the professions is always going to make such a “bad apple” approach insufficient. We can therefore consider that the profession itself may be a “bad barrel” (lurking in a poorly lit part of the “cellar”), and we may need to respond accordingly. In this regard, recent reforms of the self-regulatory process such as the increase in lay oversight, and creeping rationalisation and standardisation have served to dilute professional control and circumscribe professional autonomy and behaviour: yet professional autonomy and dominance may be reasserted in other ways through various restratification processes involving doctors laying claim to management and oversight functions. In this way, the profession is not merely reacting to external events but also attempting to control the direction of change and actively shape the policy and implementation agenda. But whether these institutional changes, taken together, have attenuated (or exacerbated) the potential for professional wrongdoing in the NHS (and the ability of the profession to police miscreants) is unclear.

Third, there is an implicit assumption in professionalism that individuals are “knights not knaves” (LeGrand, 2003). But we can ask: how does the profession find those who have knavish tendencies, and perhaps as important, how do the professional arrangements of selection, education, training, licencing, validation, etc., along with softer systems of socialisation, provide encouragement for continuing “knightish” behaviours? Does the barrel protect or spoil the apples contained? Indeed, we may ask: what is the role of the profession in growing robust “apples” in the first place (seeing the medical profession then as both orchard *and* barrel). Moreover, the “evolving ecology” view suggest that overall systemic arrangements may not necessarily be optimum (the “bad cellar” analogy): there are new competing (market, bureaucratic and professional) logics that vie for professional attention and shape behaviour; there are boundary disputes between professionally led structures and other agencies, with scope for gaps, conflict and overlaps; and the medical profession has other “projects” beyond safeguarding from wrongdoing (indeed, sometimes in conflict with this goal).

Finally, formal Inquiries have to-date failed to get to grips with this new ecology and the ramifications in any meaningful way: their focus on organisations and systems in inquiry procedures and recommendations, while helpful in some ways, has led to other important areas being overlooked; the micro focus on bad apples similarly misses important (profession-related) meso-level organisational dynamics (including organisational strategies, structures, incentives and cultures) which shape professional behaviours, for good or ill. Inquiries, then, can be seen as contributing to the proliferation of accountabilities that has diluted the grip of self-regulation and contributed to the individual-collective tension in delivering accountability (Goodwin, 2018), yet there remain important deficits in our understanding about how the medical profession itself can properly be held to account.

In sum, while we cannot eliminate misconduct, we can recognise that problems (and their solutions) can arise at four levels (apple, barrel, cellar and orchard). Professional misconduct has a greater chance of occurring when there are design faults and operational failings at each of these levels. Moreover, the widest examination of professional failings (i.e. public inquiries), at least as presently constituted, are a weak and expensive institutional instrument for addressing failures of the regulatory apparatus. Their enduring appeal may be more symbolic than instrumental in that they allow space for public catharsis and help maintain political legitimacy for the NHS, while leaving untroubled professionally led and professionally dominated regulatory arrangements.

## Figures and Tables

**Table I:** Summarising the key Inquiries on doctor wrongdoing, from 2000

Inquiry	Context	Individual doctors’ shortcomings – from incompetence to criminality (bad apples)	Organisational setting and/or cultures (bad barrels)	Overall health system concerns (bad cellars)	Additional comments drawn from Inquiry reports
Kennedy (2001)	Public inquiry, set up in 1998, to examine the care of children receiving complex cardiac surgical services at the Bristol Royal Infirmary between 1984 and 1995	Incompetence: focussed on the high mortality rates of two surgeons This is “not an account of bad people” but a “tragedy born of high hopes and ambitions and peopled by dedicated hard-working people” Both surgeons and the hospital CEO (also, and unusually, a doctor) were found guilty of serious professional conduct by the GMC in 1998 The whistle-blower (an anaesthetist) found himself unemployable in the NHS; he left the UK in 1995 to work in Australia	“Club culture” and “culture of fear” both noted Local data indicating high levels of mortality was ignored inside the hospital Structural and individual elements of hierarchies prevented open discussion	Local data were shown, *inter alia*, to: the later President of the Royal College of Anaesthetists; the (local) Director of Anaesthetics; the Medical Director of a neighbouring hospital; and (indirectly) to the President of the Royal College of Surgeons of England, as well as two Senior Medical Officers at the Department of Health Other concerns included “territorial loyalties and boundaries within the culture of medicine and the NHS, and also the realities of power and influence”	Although there were flaws with the hospital, its organisation and culture and the wider NHS, there were individuals who “should and could” have behaved differently
Ayling (2004)	Originally announced in 2001, but change of chair announced in 2002	Criminality: GP and hospital doctor, Clifford Ayling, was arrested and charged in 1998 with indecently assaulting former patients In 2000 he was convicted on 12 counts of indecent assault, relating to ten female patients, and sentenced to four years’ imprisonment. His name was placed indefinitely on the sex offender’s register; the GMC then determined that Ayling’s name should be erased from the Medical Register Inquiry identified a number of missed opportunities from 1971 until 1998: “individuals who could and should have acted on the information then available”	No investigation occurred after an incident was reported in 1980 Incident was brought to the attention of the health authority in 1991, but was not taken sufficiently seriously by senior management. Culture that saw complaints as a challenge, rather than a source of information and an opportunity to learn	Local medical committee and Medical Defence Organisation were aware of concerns, but no actions taken Health Authority finally alerted police in 1998	The single most important barrier was the absence of any formal procedure for reporting concerns about criminality
Neale (2004)	In 2000, the General Medical Council erased Richard Neale’s name from the Register	Criminality Richard Neale was erased from the Canadian Medical Register in 1985, but was subsequently allowed to work in the UK Neale then cautioned by police for an incident at a public toilet; investigation in 1993 resulted in demotion. Subsequent disciplinary hearing in 1995 led to a negotiated severance package. However, Neale then employed by two other hospitals in NHS “Alert Letter” not sent out by NHS Regional Office to NHS Health Authority and Trust Chief Executives until 1998	Culture of complacency (“We have no complaints”) that would make it extremely difficult for patients to raise concerns An urgent need for a root and branch change in attitudes and culture within the NHS	Systems failures within the employment and complaints procedures within the NHS between 1985 and 1997, and very importantly, failures within other professional bodies upon whom the NHS was dependent The most perplexing aspect was how Neale could be struck off in Canada, but able to retain his licence to practise medicine in the UK (GMC)	Both the system and those operating in it were not operating as effectively as they should have been to guarantee patient confidence and patient safety
Kerr/ Haslam (2005)	Sexual abuse of psychiatric patients in hospitals by two doctors, William Kerr and Michael Haslam In 2000 Kerr was convicted (in his absence, on a Trial of the Facts) of one count of indecent assault, and in 2003 Haslam was convicted of four counts of indecent assault (a conviction of rape was quashed on appeal)	Criminality Many ignored warning bells or dismissed rumours and some chose to remain silent when they should have been raising their voices Some stepped forward, but those lone voices were not heard Above all it was an account of psychiatric patients, whose concerns and complaints fell on deaf ears Many staff turned “a blind eye” Rumours of Kerr’s alleged abuse of female patients were well known to GPs in Harrogate, but only in later years with a “change of culture” that GPs reported In 1964 a GP practising in Northern Ireland (Mathewson), ignored pressure that he should not give evidence against a colleague, and pursued a complaint by a young female patient against Kerr, resulting in the ending of Kerr’s career in Northern Ireland: “It is a sad fact… that once in England there was not a single GP who displayed the fortitude of Dr Mathewson”	A story of management failure, failed communication, poor record keeping and a culture where the consultant was all-powerful Internal Inquiry entirely excluded the whistle-blower’s allegation of sexual abuse, and concentrated on the messenger and the substance of the message was both lost and ignored So-called “unhealthy” culture where professionals were reluctant to take action against consultants Concluded that change of culture is at the heart of real change	In 1997, NHS Regional Office set up an Enquiry into the allegations of sexual misconduct against Michael Haslam between 1984 and 1988, who was dismissed from his post in 1998 Whatever the systems in place, if those who operate them at all levels are not focussed on patient safety, then other factors, other pressures, will prevail	Key factors explaining GPs’ lack of response: the old-boy network or professional loyalty; the isolation of GPs; tolerance of sexualised behaviour; insufficient expertise in psychiatry; confidentiality; the power of consultants; and an ambivalent attitude to relationships between doctor and patient Stressed the importance of individual “agency” explanations, as opposed to collective, structural factors
Shipman (2005)	Harold Shipman was a GP who was convicted in 2000 of murdering 15 patients, and of forging a will. However, the Inquiry identified 215 victims, but “the true number is far greater and cannot be counted” In 1976 Shipman had been convicted of offences of forgery, of unlawful possession of pethidine and of obtaining pethidine by deception, but was allowed to return to unsupervised general practice in 1977	Criminality In 1994, Shipman gave a gross overdose of diamorphine to a 46-year-old patient, who later died in Tameside General Hospital. The report noted that both consultants in charge who did not report the event “must be criticised” for their failure to report Shipman’s actions, but that this is “tempered” because the culture within the profession at the time was that to report a colleague was “not done”, and many doctors throughout the country would have failed to act, as these two doctors did	The culture was that it was “not done” to report a colleague, and even today, after the Kennedy Inquiry, that culture survives in some quarters The culture in medicine inhibited the proper reporting of concerns by nurses about doctors	Smith ended her review of the Shipman case by stating she was “driven to the conclusion that, for the majority of GMC members, the old culture of protecting the interests of doctors lingers on”. The GMC, she said, was “doctor-centred”. It appeared to assume that all doctors were good, competent and conscientious until proved otherwise. It would deal with the profession’s “bad apples” for the sake of the profession [but] it would do so in its own way and it did not welcome scrutiny	Contributory factors included: fear of accusations of disparagement; the insufﬁciency of evidence; ignorance of procedures; fear of being seen as a troublemaker; fear of recriminations or reprisals; a concern that making a report might lead to proceedings for defamation; a feeling of impotence and that nothing will be done
Francis (2010)	Concerns about high mortality rates at the Mid Staffordshire NHS Foundation Trust, and an increasing public outcry led by a group of patients and patients’ relatives who had had experiences of poor care Resulted in an investigation by the Healthcare Commission (HCC), which published a highly critical report in March 2009, followed by two reviews commissioned by the Department of Health	Incompetence Deficiencies in staff performance and governance Evidence of a worrying acceptance of poor care, of poor behaviour among colleagues being condoned and of potentially dishonest behaviour being tolerated or even encouraged Insufficient attention to the maintenance of professional standards Some of the treatment of elderly patients could properly be characterised as abuse of vulnerable persons	The culture of the Trust was not conducive to providing good care for patients or providing a supportive working environment for staff An organisational culture which included: a culture of bullying; target-driven priorities; disengagement from management; low staff morale; isolation; lack of candour; acceptance of poor behaviours; reliance on external assessments; and denial The most important remedying factor is the fostering of “a culture of openness, self-criticism and teamwork”	Report focussed on Mid-Staffordshire Trust, but: Local confidence in the Trust and the NHS is unlikely to be restored without some form of independent scrutiny of the actions and inactions of the various organisations to search for an explanation of why the appalling standards of care were not picked up	Organisational culture of the Trust regarded as most important factor
Francis (2013)	The Conservative Secretary of State for Health in the Coalition government, Andrew Lansley, decided that this Inquiry should be a public inquiry under the Inquiries Act 2005	Incompetence The primary responsibility for allowing standards at an acute hospital trust to become unacceptable must lie with its Board, and the Trust’s professional staff	Reported a culture of fear in which staff did not feel able to report concerns; a culture of secrecy in which the trust board shut itself off from what was happening in its hospital and ignored its patients; and a culture of bullying, which prevented people from doing their jobs properly Primarily caused by a serious failure on the part of a provider Trust Board. Above all, it failed to tackle an insidious negative culture involving a tolerance of poor standards and a disengagement from managerial and leadership responsibilities A culture “focused on doing the system’s business – not that of the patients”	Second and third volumes of the Inquiry are concerned with the wider NHS The NHS system includes many checks and balances which should have prevented serious systemic failure of this sort A system that ought to have picked up and dealt with a deficiency of this scale failed in its primary duty to protect patients and maintain confidence in the healthcare system The extent of the failure of the system shown in this report suggests that a fundamental culture change is needed Aspects of a negative culture have emerged at all levels of the NHS system	Primary cause: failure of the Trust Board Secretary of State for Health in announcing inquiry: this was a failure of the Trust first and foremost, but it was also a national failure of regulatory and supervisory system As in the first inquiry, the evidence shows that an unhealthy and dangerous culture pervaded not only the Trust, but also the system of oversight and regulation as a whole and at every level Echoes of the cultural issues found in Stafford can be found throughout the NHS system
